# What do physiotherapists do in managing urinary incontinence in women in primary health care? a scoping review protocol

**DOI:** 10.3389/fgwh.2025.1561435

**Published:** 2025-06-26

**Authors:** Giovanna Campos Santos, Amanda Damasceno de Souza, Mara Lisiane de Moraes dos Santos

**Affiliations:** ^1^ Postgraduate Programme in Family Health, Integrated Health Institute, Federal University of Mato Grosso do Sul, Campo Grande, Brazil; ^2^ Postgraduate Programme in Information and Communication Technology and Knowledge Management (PPGTICGC), FUMEC University, Belo Horizonte, Brazil

**Keywords:** physiotherapy, primary health care, research protocol, scoping review, urinary incontinence, physical therapists, scope of practice

## Abstract

Urinary incontinence (UI), defined as the involuntary loss of urine, is highly prevalent among women and has a significant impact on physical, emotional, and social well-being. Although pelvic floor muscle training is widely recognized as a first-line intervention for mild to moderate UI, the role of physiotherapists in managing this condition within Primary Health Care (PHC) remains insufficiently explored. Considering the centrality of PHC in health systems and the predominance of generalist and multiprofessional teams in this setting, mapping conservative, low-complexity physiotherapeutic interventions is essential. This article presents a protocol for a scoping review aimed at identifying, examining, and synthesizing the scientific literature on physiotherapeutic practices for the management of UI in women within PHC. The review will follow the Joanna Briggs Institute (JBI) methodology and the PRISMA-P checklist. Eligible sources include full-text peer-reviewed articles, theses, dissertations, and clinical guidelines, with no publication date restrictions. Abstracts, opinion pieces, unrelated studies, and duplicates will be excluded. Additional strategies such as interlibrary loan services and author contact will be employed to access full texts. This review is expected to identify existing practices, knowledge gaps, and opportunities for strengthening physiotherapy care in PHC, contributing to improved health outcomes and future research directions.

## 1 Background

Urinary incontinence (UI) is defined by the International Continence Society as any involuntary loss of urine (1, 2). It is classified into three main types: (a) stress urinary incontinence (SUI)—the loss of urine associated with increased intra-abdominal pressure; (b) urgency urinary incontinence—the loss of urine accompanied by a sudden, compelling urge to void; and (c) mixed urinary incontinence—a combination of both types. SUI is the most prevalent form, accounting for approximately 60% of cases (2). This type occurs when intra-abdominal pressure exceeds the resistance provided by the pelvic floor muscles (PFM) to the urethral sphincter (3).

UI affects women more frequently, with an incidence two to four times higher than that observed in men—a difference that tends to increase with advancing age (4). Population-based studies conducted in various countries have reported UI prevalence rates ranging from approximately 5% to 70%, with most studies indicating that the prevalence of any type of UI lies between 25% and 45% (4). Studies conducted in different European countries report prevalence rates ranging from 25% to 44% among women (5). Additionally, a global systematic review estimated a mean prevalence of 37.1% of UI among older women, with the highest rates recorded in Asia (45.1%) and the lowest in the Americas (25.8%) (6).

Several risk factors are associated with UI in women, including heavy physical labor, obesity, type 2 diabetes, and chronic constipation (7–10). Studies have shown that obese women or those with diabetes have approximately twice the risk of developing UI compared to women without these conditions (7). Severe constipation, particularly when accompanied by repeated straining during bowel movements, is also a significant risk factor (9, 10). The number and type of childbirths may influence the occurrence of UI, although not all studies confirm this association (11). A systematic review with international scope supports these findings, identifying the following as the main factors associated with UI incidence among older women: advanced age, obesity, diabetes, low educational attainment, multiparity, hypertension, smoking, and urinary tract infections (12).

The impact of UI on quality of life is well documented (13), with established associations with anxiety and depression (14), as well as psychosocial, emotional, and hygienic repercussions that interfere with daily living activities (15). Shame and stigma related to the condition serve as significant barriers to treatment-seeking (16). UI also affects occupational performance, resulting in reduced productivity, changes in work routine, and challenges in maintaining employment (4, 17).

Given its high prevalence, associated treatment costs, and consequences for functionality and well-being, UI is regarded as a significant public health issue (15), imposing substantial economic burdens on both individuals and healthcare systems (16). Direct costs include medical consultations, therapies, medications, surgical procedures, and supplies such as absorbent products; indirect costs encompass productivity loss, absenteeism, and early retirement (17). In this context, adopting effective and cost-efficient therapeutic strategies is a strategic necessity to mitigate the condition's impact on health and public resources.

Therapeutic options for UI fall into three main categories: surgical, pharmacological, and conservative interventions (18). Among conservative interventions, pelvic floor muscle training (PFMT) is widely recommended as a first-line treatment due to its positive clinical outcomes, low cost, and absence of significant adverse effects (19–21). Other techniques include biofeedback, electrical stimulation, vaginal cones, and bladder training. PFMT has demonstrated particular effectiveness in cases of mild to moderate SUI (19–21). Factors such as lower severity and shorter duration of symptoms favor better outcomes (22, 23), whereas a history of pelvic surgeries may compromise the therapeutic response (22). Nevertheless, PFMT is still recommended even in cases with reserved prognosis, as treatment failure cannot be reliably predicted (24).

Physiotherapy is well established as a safe, effective, and cost-efficient therapeutic approach for managing UI. A systematic review (18) assessing the efficacy of non-pharmacological interventions in women aged 40–65 years highlighted that physiotherapists were the most frequently mentioned professionals in conducting treatments. The results demonstrated that PFMT, whether used alone or in combination with other therapeutic strategies, led to either complete resolution of UI or significant symptom improvement when compared to control groups or alternative standalone therapies. Owing to its low cost, favorable safety profile, and compatibility with other care modalities, physiotherapy stands out as one of the main recommended interventions for women with UI in the context of Primary Health Care (PHC) (25). Nevertheless, its integration remains limited, and there are still important knowledge gaps regarding its effective implementation within PHC settings (26). Thus, it is essential to explore and map existing practices and to describe the scope of physiotherapeutic interventions in this context.

Internationally, there is a growing movement to strengthen rehabilitation within Primary Health Care (PHC), led by the World Health Organization (27), aiming to overcome the notion that physiotherapeutic interventions should be limited to specialized levels of care (28). This perspective is often reinforced by the belief that physiotherapy represents a high-cost intervention and, therefore, should be restricted to secondary and tertiary services (29). However, evidence indicates that a significant portion of cases currently referred to specialized care could be effectively managed by physiotherapists within PHC, ensuring both satisfactory clinical outcomes and reduced costs (30).

Within this framework, PHC emerges as the backbone of the healthcare network, coordinating the various levels of care and enabling early, continuous, and community-based interventions. This approach reduces economic and geographical barriers, enhances adherence to treatment in the medium and long term, and strengthens the therapeutic bond between users and care teams (27, 29–32). The consolidation of this model should be regarded as a strategic investment, as it expands access to services, promotes improvements in functionality and quality of life, and consequently contributes to reducing the social and economic costs borne by both individuals and healthcare systems (31, 32).

In this context, conducting a scoping review on physiotherapeutic practices targeting women with UI within PHC is essential to map the interventions being implemented at this level of care, identify gaps in the literature, and inform the development of future research and public policies. A search conducted in the MEDLINE, PROSPERO, and JBI Evidence Synthesis databases (August 2024) did not identify any completed or ongoing systematic or scoping reviews on this topic. This is, therefore, the first scoping review dedicated to analyzing physiotherapeutic practices in the management of female UI in Primary Health Care. The methodology adopted will follow the Joanna Briggs Institute (JBI) guidelines (33), beginning with the development of a protocol designed to minimize bias during the search and study selection processes, ensure consistency among reviewers, and uphold the rigor and reproducibility of each methodological stage (32, 34).

Therefore, the aim was to develop a scoping review protocol to map, examine, and synthesize the literature on physiotherapeutic practices directed at the care of women with UI within PHC, contributing to the consolidation of knowledge in the field and to the improvement of physiotherapeutic care provided at this level of the health system.

## 2 Methods

This is a scoping review protocol designed to systematically map the available literature on the scope of physiotherapeutic practices directed at the care of women with urinary incontinence (UI) within Primary Health Care (PHC).

The review will be conducted in accordance with the Joanna Briggs Institute (JBI) methodology for scoping reviews, following established methodological references (33–35). The present protocol was developed in compliance with the EQUATOR Network (Enhancing the QUAlity and Transparency Of health Research) methodological guidelines and adheres to the PRISMA-ScR (Preferred Reporting Items for Systematic Reviews and Meta-Analyses extension for Scoping Reviews) (33), as well as the framework proposed by Peters et al. (34), in order to be guided by the principles of transparency, consistency, and methodological reproducibility in scoping reviews. The protocol is registered with the Open Science Framework (https://osf.io/k8qht/).

Based on the guiding question, “What is the scope of physiotherapeutic practices (assessment and interventions) in the treatment of women with urinary incontinence in Primary Health Care?”, and using the PCC strategy (Population, Concept, and Context), a search strategy combining controlled descriptors and keywords was developed. This combination followed the search parameters of each electronic database and was based on a predefined mnemonic framework. Search terms were then grouped using Boolean operators “AND” and “OR.”.

For searches conducted in MEDLINE and other databases, both Medical Subject Headings (MeSH) and natural language terms were used. For searches in Portuguese, Descritores em Ciências da Saúde (DeCS) were employed to ensure comprehensive coverage (Supplementary Appendix).

### 2.1 Selection of studies/sources of evidence

Following the guiding research question and the PCC strategy, the combination of controlled descriptors and keywords was defined according to the specific requirements of each electronic database and aligned with a predefined mnemonic. The inclusion criteria adopted for this review include: full-text publications available for access and analysis; studies addressing the role of physiotherapy in the management of female urinary incontinence in the context of PHC; and studies published without time restrictions, provided they address the objectives of this review. Conversely, the exclusion criteria will comprise: studies focusing on populations who have undergone surgical procedures for UI correction or those with a history of gynecologic cancers, such as uterine cancer; studies that exclusively target populations with neurological conditions affecting urinary control, such as spinal cord injuries or multiple sclerosis; and research that does not describe specific physiotherapeutic interventions or does not present outcomes related to the effectiveness of such interventions in PHC. Furthermore, abstracts, letters to the editor, opinion articles, studies that fail to meet the inclusion criteria, and duplicate records across databases will also be excluded.

Abstracts were excluded due to their insufficient detail regarding the variables necessary for data extraction, which are essential for meeting the objectives of this review. To obtain full texts for the selected studies, additional strategies will be employed, including interlibrary loan services through Brazilian and international networks and direct contact with authors when articles are not publicly available. If these approaches prove insufficient, articles deemed relevant based on abstract screening may be purchased when necessary to ensure access to the required content for critical appraisal and potential inclusion in the review.

It is important to note that the possibility of acquiring paid articles was not included in the original protocol registered on the OSF platform. This amendment was incorporated into the manuscript in response to a reviewer's recommendation, who highlighted the risk of selection bias when limiting inclusion to open-access studies. The revision aims to enhance the rigor and comprehensiveness of the mapping process while maintaining previously established methodological standards.

The evidence selection process involved identifying health-related terms using DeCS and MeSH relevant to the research topic (Supplementary Box 1). Various search strategies will be employed, applying Boolean operators “AND” and “OR” (Supplementary Box 2). English-language descriptors will be used in international databases, while terms in English, Spanish, and Portuguese will be used for searches in Latin American and Caribbean databases. The objective is to map physiotherapy practices in PHC for women with UI in a global context. Reference management will be conducted using Rayyan software. Additionally, a librarian will oversee the search strategy to ensure methodological rigor throughout the review process.

Initially, searches will be conducted in the following databases: Latin American and Caribbean Health Sciences Literature (LILACS), PubMed, EMBASE, SciVerse Scopus (SCOPUS), Scientific Electronic Library Online (SciELO), Cochrane Central Register of Controlled Trials, and PEDro.

Regarding grey literature, the following resources will be consulted: the Brazilian Coordination for the Improvement of Higher Education Personnel (CAPES) Thesis and Dissertation Catalog (https://catalogodeteses.capes.gov.br/catalogo-teses/), and Google Scholar (Supplementary Box 3).

To ensure that no similar scoping reviews or protocols existed, a search was conducted in August 2024 in the JBI, PROSPERO, and Cochrane Library databases. As no similar scoping reviews were identified, this review was initiated accordingly.

#### 2.1.1 Population

The target population—adult women aged 18–75 years with idiopathic urinary incontinence—was defined in recognition of the high prevalence of this condition across various age groups and life contexts. Among older women, UI is associated with factors such as aging, menopause, vaginal deliveries, and pelvic floor muscle weakening, affecting up to 43% of women between the ages of 35 and 81 (19, 37). Additionally, UI among younger women—often linked to high-impact activities such as sports—has become an emerging concern, with prevalence rates reaching up to 20% in this age group (38, 39). This broad prevalence highlights the need for targeted interventions for specific subgroups within PHC settings.

#### 2.1.2 Concept

The scope of physiotherapeutic practices in the treatment of UI encompasses a range of effective, evidence-based interventions. Pelvic Floor Muscle Training (PFMT) stands out as the primary first-line therapeutic approach due to its safety and efficacy, especially in cases of mild to moderate stress urinary incontinence (19). Moreover, complementary modalities such as biofeedback, vaginal cones, electrical stimulation, and bladder training provide additional therapeutic options tailored to the individual needs of patients (19). Continued research on these interventions is essential to optimize outcomes and expand therapeutic possibilities within the PHC context.

#### 2.1.3 Context

Primary Health Care (PHC) is a critical setting for the management of urinary incontinence, serving as the main access point to health services for many women. However, physiotherapeutic practices at this level face challenges related to resource availability, professional training, and the implementation of evidence-based care. The use of low-complexity technologies, such as PFMT, aligns with the principles of PHC and represents a promising strategy for managing mild to moderate cases of UI.

### 2.2 Data analysis and presentation

At this stage, the previously outlined search strategies will be employed to conduct the literature search and data collection. To strengthen the study's methodological rigor, a pilot test will be conducted in which two reviewers will independently evaluate the titles and abstracts of 10 randomly selected manuscripts. The aim is to achieve at least 75% agreement based on the previously established inclusion criteria.

Following the pilot phase, the identified studies will undergo an independent screening process conducted by two researchers. This initial screening will consist of assessing titles and abstracts against the pre-established inclusion criteria. If the abstract does not provide sufficient information to determine inclusion or exclusion, the reviewers will assess the full text. Studies that fail to meet the predefined eligibility criteria will be excluded.

Studies that meet the inclusion criteria based on this initial screening will proceed to full-text review, aiming to confirm whether the study effectively addresses the research question. Articles that do not meet this requirement will be excluded from the review process.

In cases where there is disagreement between reviewers regarding study selection, efforts will be made to reach consensus. If the disagreement persists, a third reviewer will be consulted, and their decision will be considered final for the inclusion or exclusion of the study (22).

The selected manuscripts will be obtained in full to enable independent data extraction by the two reviewers, who will remain blinded to each other's assessments throughout the evaluation process. The search results will be graphically represented using a flow diagram in accordance with PRISMA-ScR guidelines (34).

For data consolidation, an Excel spreadsheet will be used to extract and organize the following information from each study: study identification number (ID), title, authors, year of publication, scientific journal, document type (e.g., original article, review, or thesis), language, country of origin, database where the study was retrieved, target population, relevance to the PHC context, description of assessment strategies, variables analyzed, interventions performed, and whether the results align with the objectives of this review. These criteria will enable an objective evaluation of whether the study addresses the core elements of the research question.

### 2.3 Data analysis and synthesis

The analysis of the results will include both qualitative and quantitative data, depending on the methodological nature of each study. To ensure transparency and methodological rigor, a standardized data extraction tool will be used. This instrument will include bibliometric indicators and elements structured according to the Population, Concept, and Context (PCC) framework, aligned with the objectives of this scoping review (Supplementary Box 4). Its purpose is to gather detailed information that enables a critical and interpretive synthesis of the findings, including the following: study identification, full title, objectives as stated by the authors, methodological design and nature of the study (quantitative, qualitative, or mixed), inclusion and exclusion criteria, sample size and profile, study location and contextual setting, assessment tools or strategies used, variables investigated, types of interventions described, and outcomes measured. Additionally, it will capture the main results, strengths and limitations highlighted by the authors, and any recommendations with potential implications for clinical practice and public policy.

The data will be presented in tables, charts, and/or figures, as appropriate, and summarized narratively to facilitate understanding of the topic under investigation. The data synthesis will follow the structure outlined in the review protocol. It is important to note that the data extraction tool may be refined during the selection and analysis process, with any modifications to be reported in the final version of the review.

Furthermore, the synthesis of evidence will be guided by the *Checklist for Textual Evidence: Narrative* from the JBI, a methodological tool that supports the critical analysis and structured presentation of textual and opinion-based evidence (35).

## 3 Discussion

The development of this scoping review protocol required a rigorous methodological approach, particularly in defining a research question that is both comprehensive and focused. The adoption of the Joanna Briggs Institute (JBI) methodology (33), combined with the use of the PCC mnemonic (Population, Concept, Context), provided a solid framework for identifying, selecting, and analyzing relevant studies. This ensured methodological coherence and minimized bias throughout the review process. Applying these criteria will allow for a systematic understanding of the scope of physiotherapy practice in the management of urinary incontinence (UI) within Primary Health Care (PHC), as well as highlight existing gaps in the scientific literature on this topic.

This initiative aligns with global commitments established under the Sustainable Development Goals (SDGs), particularly the goal of achieving Universal Health Coverage. These commitments call for the strengthening and qualification of Primary Health Care, recognized as the backbone of more equitable, effective, and financially sustainable health systems (36). Within this context, mapping and qualifying feasible physiotherapeutic interventions in PHC is a strategic approach to expanding access, reducing inequities, and consolidating public policies that respond to the concrete needs of populations. This effort becomes even more urgent in light of persistent health inequalities, which disproportionately affect women in vulnerable situations and other historically underserved groups.

Aligned with this context, it is important to emphasize that this protocol is grounded in the realities of PHC, where care is provided by generalist professionals and multidisciplinary teams, without the guaranteed presence of specialists. Thus, the physiotherapeutic practices considered in this protocol correspond to conservative, low-complexity interventions with the potential for effective implementation at the first level of care. These are in alignment with the principles of comprehensive care and the strengthening of health care networks.

The implementation of this protocol addresses a highly prevalent condition with significant impact on the health of adult and older women—urinary incontinence (UI)—which compromises the physical, emotional, and social dimensions of quality of life. In this context, the growing body of evidence regarding physiotherapeutic management, particularly within the PHC setting, has the potential to enhance care, expand access to low-cost conservative therapies, and increase the problem-solving capacity of PHC.

Interventions such as Pelvic Floor Muscle Training (PFMT) have shown significant efficacy in symptom reduction and functional recovery, as demonstrated by Al Belushi et al. (37), Dumoulin et al. (19), García-Giralda et al. (39), Trapani et al. (39), and Vaz et al. (40). The literature indicates that early implementation of these approaches—especially in initial stages and in mild to moderate cases of UI—contributes to slowing disease progression, preventing surgical interventions, and substantially improving the quality of life of women with UI. The study by Vaz et al. (40) revealed that home-based pelvic physiotherapy programs, structured with initial in-person guidance and remote follow-up, were effective in reducing urinary leakage episodes and improving functionality in women receiving care in PHC. The intervention consisted exclusively of PFMT exercises and was compatible with PHC resources and guidelines, demonstrating high adherence and a positive impact on participants' quality of life. Similarly, Al Belushi et al. (37) showed that unsupervised home-based PFMT, when combined with initial educational instruction and weekly remote reinforcement, significantly improved the severity of stress urinary incontinence symptoms and participants' quality of life. Together, these studies support the feasibility and effectiveness of PFMT as a conservative, accessible, and safe therapeutic strategy aligned with PHC principles and adaptable to various contexts.

Another relevant aspect is the need to consider associated conditions, such as a history of prior surgeries for pelvic organ prolapse or genital dystopia correction, which may coexist with UI and influence therapeutic approaches (41). Proper screening and identification of such factors in PHC are essential for determining whether conservative management is appropriate or referral to other levels of care is necessary.

Systematic reviews conducted by Dumoulin et al. (19) and Trapani et al. (39) further emphasize that PFMT and pelvic physiotherapy are first-line interventions for women with different types of UI, promoting symptom improvement or resolution with high safety, acceptability, and cost-effectiveness. These findings strengthen the recommendation and feasibility of incorporating such practices into the scope of PHC services.

Nonetheless, despite the robust scientific support for conservative UI treatment via physiotherapy—with PFMT widely recommended as the first-line therapy for women with UI (19)—structural and sociocultural challenges continue to limit its reach. Barriers such as restricted access to services, low treatment adherence, lack of follow-up after therapy, and the stigma and shame associated with UI significantly reduce care-seeking behavior.

In light of this, PHC presents itself as a strategic and promising setting for addressing these barriers by offering accessible, continuous, and comprehensive care centered on the individual and her social and territorial context. Providing PFMT in PHC, whether alone or in combination with other therapeutic strategies, represents a feasible, effective, and low-cost alternative compatible with the core principles of primary care. Its essential attributes—first-contact access, continuity, comprehensiveness, and care coordination—are complemented by derivative attributes such as family orientation, community engagement, and cultural competence (42, 43), all of which enhance the quality of care in ways that are responsive to the needs of women and their communities.

Moreover, physiotherapy in PHC is already a well-established reality in various health systems worldwide and serves as a key strategy to expand access to conservative UI treatment, especially in contexts of greater social vulnerability. This approach fosters humanized, community-based care that is grounded in women's lived realities and takes into account the social, cultural, and emotional determinants that directly influence treatment adherence. Educational strategies integrated into physiotherapeutic interventions—such as promoting self-care and sexual health—have also proven effective in enhancing overall well-being (44, 45). Additionally, Panicker et al. (46) highlight the relevance of psychosocial and behavioral factors in the course of UI, reinforcing the need for integrated, individualized, and culturally sensitive approaches.

Despite clinical advancements and the growing consolidation of physiotherapy practice for women with UI, the literature remains limited in its discussion of the care contexts and levels of service delivery in which these interventions are feasible, effective, and accessible. There remains a significant gap regarding studies that critically explore the clinical, organizational, and contextual dimensions of physiotherapeutic care for women with UI in PHC—including variables such as the types of practices implemented, access, adherence, and the quality of care provided. Addressing this gap requires research that not only evaluates therapeutic efficacy but also incorporates analyses of the services and levels of care where treatment occurs, how these practices are implemented, and the barriers and facilitators involved in organizing and delivering care in diverse settings.

## 4 Compliance with reporting guidelines

This manuscript adheres to the guidelines of the Enhancing the QUAlity and Transparency Of health Research (EQUATOR) network, ensuring methodological rigor and transparency in the dissemination of results.

## 5 Language review

To enhance clarity and readability, the manuscript was reviewed for linguistic accuracy by a native English speaker.

## 6 Operational and practical issues

Among the operational and practical considerations guiding the development of this scoping review protocol is the need to investigate and map the scope of physiotherapists' practices within Primary Health Care (PHC), with a particular focus on assessment strategies and both preventive and therapeutic interventions for managing urinary incontinence in women. Identifying knowledge gaps in this area is also essential, considering that PHC offers a strategic and underexplored setting for strengthening and expanding care for women affected by this condition. Additionally, this protocol highlights the importance of fostering more effective integration between physiotherapists and other health professionals within PHC teams. The limited availability of global and regional data on physiotherapy practice in this context reflects a gap in the literature that this review aims to address through a comprehensive and systematic mapping of the existing scientific evidence.

## 7 Final considerations

This scoping review on physiotherapeutic practices in the care of women with UI in PHC aims primarily to map existing knowledge, identify the nature of reported practices, and highlight areas that remain underexplored. Regarding the scope of practice, it will support the strengthening of clinical care by informing the development of care protocols and best practice guidelines in women-centered PHC. In terms of gap mapping, it will contribute by identifying actions that are not yet sufficiently structured or recognized within PHC. The absence of literature on physiotherapeutic interventions in this field—such as educational actions, care pathways, or adherence strategies—will point to the need for developing strategies to overcome these challenges. Thus, this scoping review will not only organize the available knowledge but also bring visibility to areas that are overlooked in practice and research, serving as a strategic tool to foster improvements in care and public health management.
