# Supplementary material for: What do physiotherapists do in managing urinary incontinence in women in primary health care? a scoping review protocol
Source: Front Glob Womens Health. 2025 Jun 26;6:1561435. doi: 10.3389/fgwh.2025.1561435 (PMC12240972; doi:10.3389/fgwh.2025.1561435)
Supplement: Supplementary file 1 [file Table1.docx]

**Box 1.** Identification of terms DeCS and MeSH to be employed in the search strategy of scientific evidence.

| **Mneumonic** | **DeCS/MeSH** | **Identified keywords** | **Natural language** |
| --- | --- | --- | --- |
| PARTICIPANTS | Urinária  Incontinência  Urinary Incontinence | Incontinence, Urinary  Incontinencia Urinaria  Incontinence urinaire | Urinary Incontinence |
|  | Saúde da Mulher  Women's Health | Health, Woman's Health, Women's Health, Womens Woman's Health Womens Health |  |
| CONCEPT | Serviços de Saúde da Mulher  Women's Health Services | Serviços de Saúde das Mulheres  Serviços de Saúde para a Mulher  Serviços de Saúde para as Mulheres  Serviços de Saúde para Mulheres |  |
|  | Modalidades de Fisioterapia  Physical Therapy Modalities | Fisioterapia  Fisioterapia (Técnicas)  Fisioterapia em Grupo  Fisioterapia Grupal  Fisioterapia Respiratória  Fisioterapias em Grupo  Técnicas de Fisioterapia  Técnicas Fisioterápicas | Tratamento fisioterapêutico  tratamento em fisioterapia  Physiotherapy treatment  physiotherapy treatment |
|  | Reabilitação  Rehabilitation | Habilitação | physical therapy treatmen  Physical Therapeutic Exercise  Physical Therapeutic Interventions |
|  | TMAP | Treinamento da Musculatura do Assoalho Pélvico | Pelvic Floor Muscle Training |
| CONTEXT | Atenção Primária à Saúde | Atendimento Básico  Atendimento Primário  Atendimento Primário de Saúde  Atenção Básica  Atenção Básica à Saúde  Atenção Básica de Saúde  Atenção Primária  Atenção Primária de Saúde  Atenção Primária em Saúde  Cuidado de Saúde Primário  Cuidado Primário de Saúde  Cuidados de Saúde Primários  Cuidados Primários  Cuidados Primários à Saúde  Cuidados Primários de Saúde  Primeiro Nível de Assistência  Primeiro Nível de Atendimento  Primeiro Nível de Atenção  Primeiro Nível de Atenção à Saúde  Primeiro Nível de Cuidado  Primeiro Nível de Cuidados  Núcleo Ampliado Saúde da Família | Primary Health Care |
