# Supplementary material for: What do physiotherapists do in managing urinary incontinence in women in primary health care? a scoping review protocol
Source: Front Glob Womens Health. 2025 Jun 26;6:1561435. doi: 10.3389/fgwh.2025.1561435 (PMC12240972; doi:10.3389/fgwh.2025.1561435)
Supplement: Supplementary file 2 [file Table2.docx]

**Box 2 .** Search Strategy

| Database | Search Strategy |
| --- | --- |
| LILACS (via BVS)  Resultas:1 | (urinary incontinence) AND (women's health) AND (women's health services ) AND (physical therapy modalities ) OR (rehabilitation) OR (physiotherapy treatment) AND (primary health care) AND db:("LILACS") AND instance:"regional" |
| Medline (via PubMed)  Resultas:224 | (((Urinary Incontinence[Title/Abstract]) AND (Women's Health[Title/Abstract])) AND (Women's Health Services[Title/Abstract]) AND (Physical Therapy Modalities[Title/Abstract]) AND ("Physiotherapy treatment"[Title/Abstract]) OR (Rehabilitation[Title/Abstract]) OR ("Pelvic Floor Muscle Training") AND ("Primary Health Care"[Title/Abstract])) Filters: Female, MEDLINE |
| Library Online (SciELO),  Results:3 | (Urinary Incontinence AND Women AND primary Health Care AND Physiotherapy) |
| EMBASE (Elsevier)  Results: 276 | (('urine incontinence'/exp OR 'urine incontinence') AND 'women`s health':ti,ab,kw AND 'health service':ti,ab,kw AND rehabilitation:ti,ab,kw OR physiotherapy:ti,ab,kw OR 'pelvic floor muscle training':ti,ab,kw) AND 'primary health care':ti,ab,kw |
| SCOPUS (Elsevier)  Results:6 | ( TITLE-ABS-KEY ( "Urinary Incontinence" ) AND TITLE-ABS-KEY ( "Women&apos;s Health" ) AND TITLE-ABS-KEY ( "Women&apos;s Health Services" ) OR TITLE-ABS-KEY ( rehabilitation ) OR TITLE-ABS-KEY ( "Physical Therapy Modalities" ) OR TITLE-ABS-KEY ( "Pelvic Floor Muscle Training" ) AND TITLE-ABS-KEY ( "Primary Health Care" ) ) |
| PEDro  Results: 200  10 first pages | "Urinary Incontinence""Women's Health""Primary Health Care""Physiotherapy treatment" |
| Cochrane Library  Results: 497 | "Urinary Incontinence" AND "Women's Health" OR "Women's Health Services" in Title Abstract Keyword AND Rehabilitation OR "Physical Therapy Modalities" OR "Physiotherapy treatment" in Title Abstract Keyword OR "primary health care center" in Title Abstract Keyword AND "Primary Health Care" in Title Abstract Keyword - (Word variations have been searched) |
