# Supplementary material for: What do physiotherapists do in managing urinary incontinence in women in primary health care? a scoping review protocol
Source: Front Glob Womens Health. 2025 Jun 26;6:1561435. doi: 10.3389/fgwh.2025.1561435 (PMC12240972; doi:10.3389/fgwh.2025.1561435)
Supplement: Supplementary file 3 [file Table3.docx]

**Box 3:** Search strategy in gray literature

| **Search strategy** | **Gray literature**  **Number of publications** |
| --- | --- |
| ((("Urinary Incontinence") AND ("Women's Health")) AND ("Women's Health Services") OR ("Physical Therapy Modalities") OR ("Physiotherapy treatment") OR (Rehabilitation) AND ("Primary Health Care"))) | Google Scholar  Number: 337 |
| "Urinary Incontinence" AND "Physical Therapy Modalities" | Theses and Dissertations of the Coordenação de Aperfeiçoamento do Pessoal de Nível Superior (CAPES)  Number: 3 |
