# Supplementary material for: What do physiotherapists do in managing urinary incontinence in women in primary health care? a scoping review protocol
Source: Front Glob Womens Health. 2025 Jun 26;6:1561435. doi: 10.3389/fgwh.2025.1561435 (PMC12240972; doi:10.3389/fgwh.2025.1561435)
Supplement: Supplementary file 4 [file Table4.docx]

**Box 4:** Tool of extraction of bibliometric data and related to PCC

| **Article/publication/Citation** |  |  |  |  |
| --- | --- | --- | --- | --- |
| **Authors** |  |  |  |  |
| **Year/Month** |  |  |  |  |
| **Country** |  |  |  |  |
| **Idiom of publication** |  |  |  |  |
| **Objective** |  |  |  |  |
| **Method** |  |  |  |  |
| **Population** |  |  |  |  |
| **Context** |  |  |  |  |
| **Evaluation** |  |  |  |  |
| **Intervention** |  |  |  |  |
| **Results** |  |  |  |  |
| **Recommendations** |  |  |  |  |
| **Forces** |  |  |  |  |
| **Limitations** |  |  |  |  |
